# Eocene “*Chusquea*” fossil from Patagonia is a conifer, not a bamboo

**DOI:** 10.3897/phytokeys.139.48717

**Published:** 2020-02-03

**Authors:** Peter Wilf

**Affiliations:** 1 Department of Geosciences, Pennsylvania State University, University Park, PA 16802, USA Pennsylvania State University University Park United States of America

**Keywords:** Gondwana, Laguna del Hunco, Poaceae, Podocarpaceae, *
Retrophyllum
*, South America

## Abstract

*Chusquea oxyphylla* Freng. & Parodi, 1941, a fossilized leafy branch from the early Eocene (52 Ma), late-Gondwanan Laguna del Hunco biota of southern Argentina, is still cited as the oldest potential bamboo fossil and as evidence for a Gondwanan origin of bamboos. On recent examination, the holotype specimen was found to lack any typical bamboo characters such as nodes, sheaths, ligules, pseudopetioles, or parallel leaf venation. Instead, it has decurrent, clasping, univeined, heterofacially twisted leaves with thickened, central-longitudinal bands of presumed transfusion tissue. These and other features allow confident placement in the living Neotropical and West Pacific disjunct genus *Retrophyllum* (Podocarpaceae), which was recently described from the same fossil site based on abundant, well-preserved material. However, the 1941 fossil holds nomenclatural priority, requiring the new combination *Retrophyllum oxyphyllum* (Freng. & Parodi) Wilf, **comb. nov.** No reliable bamboo fossils remain from Gondwana, and the oldest South American bamboo fossils are Pliocene. *Chusquea* joins a growing list of living New World genera that are no longer included in Paleogene Patagonian floras, whose extant relatives are primarily concentrated in Australasia and Malesia via the ancient Gondwanan route through Antarctica.

## Introduction

**Figure 1.**
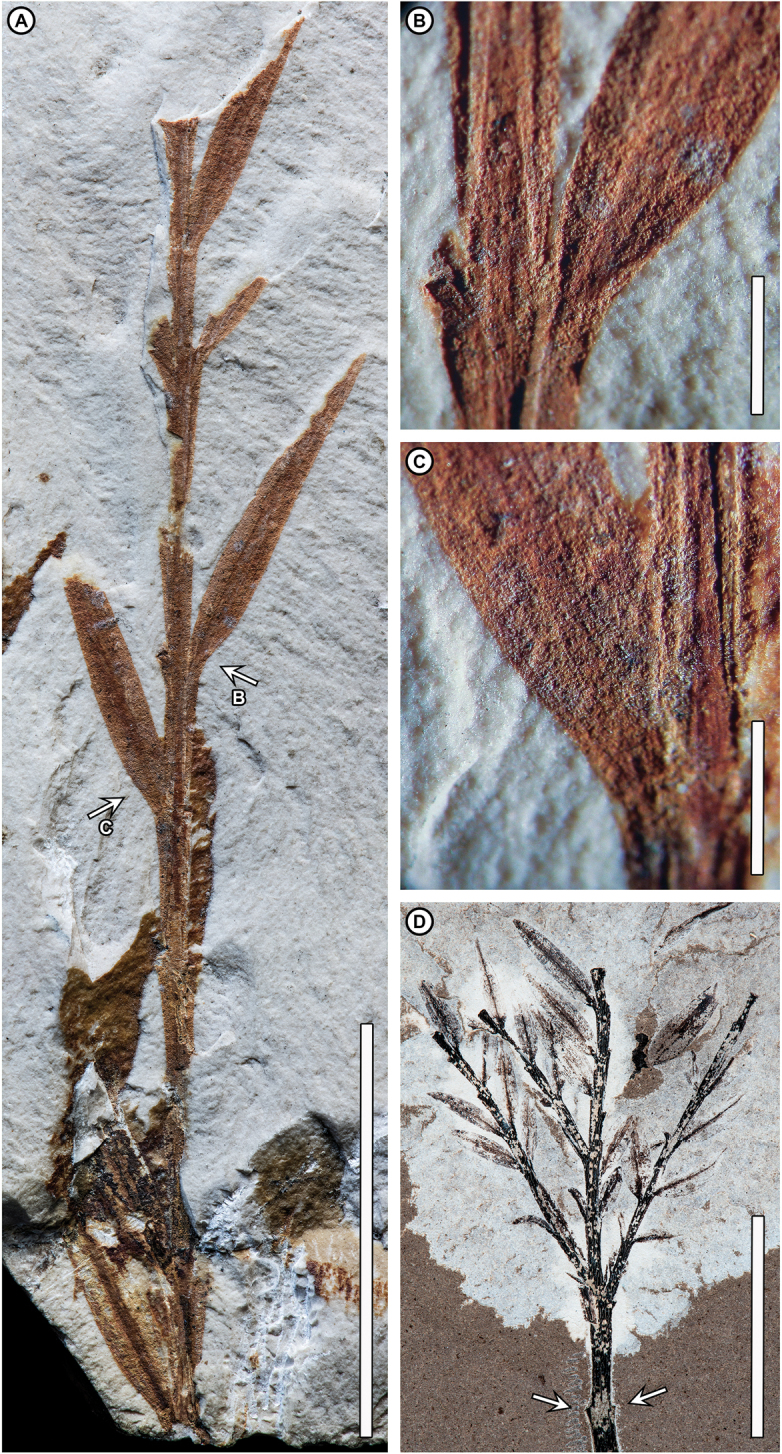
*Retrophyllum oxyphyllum* (Freng. & Parodi) Wilf, comb. nov. from Laguna del Hunco. **A–C** Holotype, MLP-4234, arrows in **A** indicate detail panels in **B, C**. **D** MPEF–Pb 8915a (Museo Paleontológico Egidio Feruglio, Trelew, Argentina) from Laguna del Hunco quarry LH6 of Wilf et al. (2003), part of an extensive suite of fossil *Retrophyllum* material here synonymized (Wilf et al. 2017b; Wilf 2017). **A** General view of the holotype, with clasping, overlapping, zigzagging, decurrent, opposite leaf bases; heterofacially twisted, lanceolate free-leaf portions, many of them broken off at departure from the twig and leaving stubs; and thick central tissue band compressed to a coalified black stripe, most conspicuous in the basalmost preserved leaf. Leaves on the left side of the twig in this view are twisted “forward” and those on the right “backward,” i.e., counterclockwise in both cases when viewed from leaf to twig; original abaxial or adaxial orientation cannot be determined **B** detail of backward-twisted leaf (at right), with negative relief (from compression) of the raised central band visible, and broken leaf base (at left) **C** detail of forward-twisted leaf, thickened central band, and dense longitudinal striations across the leaf surface marking borders between former stomatal rows **D** terminus of a long penultimate branch (also Wilf et al. 2017b: figs 10–12) with pairs of opposite, ultimate leafy branches, each similar to the holotype (A) with opposite leaves and linear-reduced leaves at the shoot bases (the holotype does not preserve the shoot base). Arrows indicate opposite branch scars on the exposed penultimate branch, completely unlike bamboo nodes. Scale bars: 2 cm (**A, D**); 2 mm (**B, C**).

In 1941, the legendary Argentine botanists Joaquín Frenguelli and Lorenzo R. Parodi of Museo de La Plata (Frenguelli and Parodi 1941; Burkart 1967; Riccardi 2017) described a compressed leafy-shoot fossil from northwest Chubut Province, Argentina under *Chusquea* Kunth, a diverse New World bamboo genus (Clark 1989, 1997a; Fisher et al. 2014; Wysocki et al. 2015). *Chusquea oxyphylla* Freng. & Parodi, 1941 (Fig. 1A–C) was one of the earliest taxonomic contributions to the extraordinarily diverse Laguna del Hunco biota (Berry 1925; Dolgopol de Sáez 1941). The assemblage, once thought to be Miocene in age, is now constrained to the early Eocene (ca. 52.2 Ma; Wilf et al. 2003, 2017a); it has remained a subject of intensive study for many decades (e.g., Romero and Hickey 1976; Fidalgo and Smith 1987; Romero et al. 1988), particularly over the past ca. 15 years (for summaries see, e.g., Wilf et al. 2009, 2013, 2019).

*Chusquea oxyphylla* retains significance today because, at 52 Ma, it is by far the oldest putative bamboo macrofossil and the only one still recognized (by some authors) from Gondwana. Otherwise, reliable South American bamboo fossils are no older than Pliocene (Brea and Zucol 2007; Olivier et al. 2009; Brea et al. 2013), making them much younger than Gondwana, whose final separation began ca. 50 Ma (e.g., Lawver et al. 2011), and contemporary with the closure of the Isthmus of Panama and direct biotic interchange with Central and North America (e.g., Simpson 1950; O’Dea et al. 2016). Worldwide, reliable bamboo macrofossils are no older than Oligocene (e.g., Worobiec and Worobiec 2005; Brea et al. 2013; L. Wang et al. 2013; Q. Wang et al. 2014; Srivastava et al. 2019). The oldest microfossil (phytolith) evidence for bamboos is from the middle Eocene of the Northern Hemisphere (Strömberg 2004, 2005, 2011). Thus, *C. oxyphylla* remains prominent, with variable confidence expressed regarding its affinities, in discussions about the age, paleoecology, biogeography, and possible Gondwanan origins of bamboos (Barreda and Palazzesi 2007; Brea and Zucol 2007; Iglesias et al. 2011; Ruiz-Sanchez 2011; L. Wang et al. 2013; Giussani et al. 2016; Srivastava et al. 2019). However, several authors have doubted that *C. oxyphylla* is a definite bamboo or even a grass (Thomasson 1980; Srivastava et al. 2019).

*Chusquea oxyphylla* has biogeographic significance for Laguna del Hunco and other Eocene Patagonian floras, which were once considered to be closely allied with extant South American floras from proximal areas such as Paraguay and northern Argentina, where *Chusquea* is a prominent element (Berry 1925; Frenguelli and Parodi 1941). In contrast, several putative New World elements from Laguna del Hunco have been revised recently to taxa whose living members primarily inhabit the Asia-Pacific region via Gondwanan connections (e.g., *Austrocedrus*-*Libocedrus* to *Papuacedrus*, *Fitzroya* to *Dacrycarpus*, *Zamia* to *Agathis*; Wilf et al. 2009, 2014; Wilf 2012). Moreover, numerous additional taxa have been described from the site that also have Asia-Pacific extant distributions (e.g., Romero and Hickey 1976; Zamaloa et al. 2006; Gandolfo et al. 2011; Carpenter et al. 2014; Gandolfo and Hermsen 2017; Andruchow-Colombo et al. 2019; Wilf et al. 2019). Also, monocots in the Laguna del Hunco flora are scarce in general, otherwise represented by a few leaves of *Ripogonum* (Ripogonaceae; Carpenter et al. 2014) and rare, undescribed palm fruits and leaf fragments (Wilf et al. 2005). The specimen referred to *Poacites* sp. Berry (1925; National Museum of Natural History, Smithsonian Institution [USNM], USNM 219072), on my examination, is too poorly preserved to assign confidently to any plant group, much less to the grasses. Despite the general significance of *C. oxyphylla*, until now there have been no published re-examinations of the holotype (Fig. 1A–C), otherwise known only from a single photograph in the original publication (Frenguelli and Parodi 1941).

## Materials and methods

I examined the holotype of *Chusquea oxyphylla* on 26 May 2019 in the paleobotanical collections of Museo de La Plata, Argentina (**MLP**), specimen MLP-4234 (Fig. 1A–C). Specimen tags indicate “Laguna del Hunco, El Mirador, Chubut” and “Mioceno,” which was formerly considered the age of the Laguna del Hunco fossil-lake beds (Berry 1925). The protologue (Frenguelli and Parodi 1941: 235–236) states that the specimen was collected in 1939 or 1940 and came from the “basal layers” (“*capas basales*”) of the lacustrine sequence now known as the Tufolitas Laguna del Hunco (Aragón and Mazzoni 1997). However, there was no general stratigraphic section and correlation of the lake beds available in the early 1940s, and Frenguelli and Parodi (1941) more likely were referring to a relative position within a local exposure rather than the full stratigraphic sequence as later understood (Petersen 1946; Aragón and Mazzoni 1997; Wilf et al. 2003). The lithology and preservation of the holotype closely resemble fossils from the horizon of what is now quarry LH4 (see Wilf et al. 2003 for coordinates), which appears to have been the site of most early collections (see Wilf et al. 2019). Quarry LH4 is well exposed at a comparatively accessible location, low on a local hill slope, where the underlying basal strata of the lake beds (subsection E of Wilf et al. 2003) are mostly lost to a local unconformity; thus, LH4 could have appeared to be near the base of the lake beds. However, LH4 actually lies in the middle of the full 170 m stratigraphic section of the Tufolitas Laguna del Hunco at Laguna del Hunco (Wilf et al. 2003) and is now confidently dated to ca. 52.2 Ma using several ^40^Ar-^39^Ar dates and paleomagnetic data from strata intercalated with the fossil quarries; in particular, an ^40^Ar-^39^Ar age on sanidine of 52.22 ± 0.22 Ma was analyzed from a tuff only 40 cm above quarry LH4 (Wilf et al. 2003, 2005, 2017a).

Photographs were taken at MLP using a Nikon D850 DSLR with an AF-S VR Micro-Nikkor 105 mm f2.8 G IF-ED lens and a Nikon circular polarizer and on a Leica M50 stereoscope with a mounted Canon Powershot S40 camera and Canon Remote Capture 2.2 software. I consulted standard botanical literature for *Chusquea* and other bamboos (McClure 1966, 1973; Clark 1989, 1997a; Stapleton 1997; Judziewicz et al. 1999; Clark et al. 2015) and for podocarp conifers (e.g., de Laubenfels 1969; Farjon 2010; Mill 2016; others cited in Wilf et al. 2017b); these references support the discussion below.

## Taxonomic treatment

### Podocarpaceae Endl., Synopsis Coniferarum: 203 (1847).

*Retrophyllum* C. N. Page, Notes of the Royal Botanic Garden of Edinburgh 45: 379 (1989) [“1988”, see Mill 2016]).

#### Retrophyllum oxyphyllum

Taxon classificationPlantaePinalesPodocarpaceae

(Freng. & Parodi) Wilf comb. nov.

F3677C74-5713-5257-AB77-C795D04F9333

Figure 1A–C

##### Basionym.

*Chusquea oxyphylla* Freng. & Parodi, Notas del Museo de La Plata, Paleontología 6: 236 (1941: fig. 1).

##### Synonym.

*Retrophyllum spiralifolium* Wilf, American Journal of Botany 104: 1350 (2017).

##### Holotype.

Argentina. Chubut Province: Laguna del Hunco, Tufolitas Laguna del Hunco, Huitrera Formation, early Eocene. Museo de La Plata (MLP), MLP-4234. Collected by J. Frenguelli 1939 or 1940 (Frenguelli and Parodi 1941: 236), precise collection location unknown. The holotype is the only specimen of the basionym.

##### Amended description.

The entire recent description of *Retrophyllum spiralifolium* Wilf, 2017 (Wilf et al. 2017b: 1350–1352), verbatim, is here denoted as the amended formal description of *Retrophyllum oxyphyllum* comb. nov. but is not reproduced here due to its length. The holotype fully conforms to the described foliage, in particular the distichous foliage form, of *R. spiralifolium*. The new combination incorporates all associated material described, illustrated, and justified previously under *R. spiralifolium* (Wilf et al. 2017b), including the distichous foliage form, helical foliage form, reduced foliage forms, and peduncle of pollen cones.

##### Diagnostic characters.

In the absence of a diagnosis of the basionym (Frenguelli and Parodi 1941), a formal amended diagnosis cannot be provided. However, the characters listed in the specific diagnosis for *Retrophyllum spiralifolium* (Wilf et al. 2017b: 1350) all now apply to *Retrophyllum oxyphyllum* comb. nov. That diagnosis (Wilf et al. 2017b: 1350) is reproduced here for ease of use, with the characters preserved in the holotype (Fig. 1A–C) indicated in bold font:

"***Foliage with conspicuous central longitudinal band of thickened tissue and obscure midvein not separating rows of stomata.****Lateral resin canals present.****Principal leaves decurrent and extensively clasping twig, free portions either distichous and pectinate, with full heterofacial flattening***, *or spirally deployed with negligible to slight basal twisting, frequently broken off to leave spirally arranged stubs of clasping portions. Leaf apices acuminate to markedly acuminate. Terminal bud protected by reduced, modified leaves. Reduced foliage also including ovoid and narrow forms on separate shoot segments and narrow miniature leaves abruptly or gradually interspersed with principal leaves along shoots. Pollen cones pedicellate, long-cylindrical, in axils of narrow reduced leaves, distichously grouped on a common peduncle.*"

##### Amended description of the holotype.

The holotype of *Retrophyllum oxyphyllum* comb. nov. (Fig. 1A–C) is a leafy branch segment of axis length 6.4 cm with remains of ca. ten pairs of opposite, distichous (pectinate), decurrent and clasping, ovate-lanceolate, bifacially flattened leaves that are heterofacially twisted into a single plane at their departure from the twig. The clasping portions of the leaves entirely cloak the twig in an overlapping, zigzag pattern. It is not possible to determine whether the preserved view is abaxial or adaxial (see Wilf et al. 2017b). The bases of the leaves’ free portions are twisted counterclockwise if viewed laterally from leaf to twig, so that pairs of abaxial and adaxial leaf faces appear in the same plane on either side of the twig. Only ca. four leaves have their free portions well preserved; most leaves are broken off at or near twig departure, leaving behind their clasping leaf bases. Free leaf portion length is to 18.0 mm, width to 2.5 mm, apices acute but not completely preserved. Leaves have no venation visible but preserve a longitudinal, raised central band of thickened, coalified tissue whose width is ca. 25% of total leaf width; the central band presumably obscures the much smaller, true midvein running within. The remaining leaf surface has numerous parallel striations on both faces, continuous across the midvein, with slight relief but no evidence of vein tissue; there are no cross-lineations that could be interpreted as cross-veins.

## Discussion

The holotype of *Retrophyllum oxyphyllum* comb. nov. (Fig. 1A–C) does not resemble *Chusquea* or any other bamboo, and all its previously noted similarities to bamboos and other grasses (Frenguelli and Parodi 1941), though reasonable at the time, are superficial. There is no evidence of bamboo-type nodes, sheaths, or ligules as initially described (Frenguelli and Parodi 1941); areas that may resemble those features consist only of the broken departure points of leaf bases diverging from the twig. The decurrent, extensively clasping leaves are quite unlike the characteristically pseudopetiolate leaves of bamboos, and the heterofacially twisted free-leaf bases do not occur, to my knowledge, in any bamboo or grass. In the grass subfamily Pharoideae, pseudopetioles characteristically twist 180° so that all leaf abaxial surfaces face adaxially (e.g., Judziewicz et al. 1999); however, this twisting is homofacial, unlike the fossil, and the leaf architecture of Pharoideae is also completely unlike that of the fossil. The lack of leaf venation in the fossil, other than a single presumed midvein obscured by thickened tissues, contrasts with bamboos, pharoids, and other grasses, which usually have one to several discrete orders of parallel veins connected by numerous, though sometimes obscure, cross-veins and no thickened or raised laminar tissues similar to those in the fossil.

On the other hand, the holotype is easily identifiable as the flip-leaved, podocarpaceous conifer genus *Retrophyllum*; it matches precisely the distichous fossil foliage form of *Retrophyllum spiralifolium*, which was described recently from a suite of 82 specimens collected from both Laguna del Hunco, including quarry LH4, and the early middle Eocene Río Pichileufú site in Río Negro Province (Wilf et al. 2017b). *Retrophyllum* is a genus of six living species of rainforest conifers that is disjunct between the Neotropics and the tropical West Pacific, as reviewed in Mill’s (2016) recent monograph. *Retrophyllum* is the only living genus that has heterofacially twisted (flip-leaved), distichous, elliptic to ovate-lanceolate free foliage precisely like that in the fossil, similarly emerging from extensively clasping, overlapping, zigzagging leaf bases below the twist point. *Retrophyllum* is univeined and amphistomatic, as the fossil is inferred to be; the stomata deploy in longitudinal rows that are distributed nearly evenly across the blade, separated by longitudinal striations (that superficially resemble veins) with no grouping into zones or interruption at the midvein (e.g., Mill 2016). Similarly, the evenly spaced longitudinal striations on both fossil leaf surfaces (e.g., Fig. 1C and similar material in Wilf et al. 2017b), once interpreted as veins (Frenguelli and Parodi 1941), mark the areas between the original stomatal rows and trend slightly obtuse to the course of the leaf margin as in living *Retrophyllum*.

*Retrophyllum* leaves also have a thickened, raised central band, consisting of wings of transfusion tissue that is more or less prominent depending on species (Gray 1962; de Laubenfels 1969). In fossil *Retrophyllum* previously described from Laguna del Hunco (Wilf et al. 2017b) and the fossil studied here (Fig. 1A–C), the transfusion-tissue band is raised and coalified to a thick black stripe of one-fifth to one-third of total leaf width, entirely unlike the slender midveins of bamboos as previously interpreted (Frenguelli and Parodi 1941). Among the prior material here synonymized (Wilf et al. 2017b) is a spectacular, long, leafless branch segment terminating in several opposite, pectinate leafy shoots (Fig. 1D; also Wilf et al. 2017b: figs 10, 11 for complete view), each of these shoots very similar to the holotype (Fig. 1A); the exposed branch has opposite leaf scars typical of *Retrophyllum*, with no bamboo-type axis segmentation or associated features such as nodes, sheaths, sheath scars, buds, or branch complements that would be clearly visible if present. The associated peduncle of pollen cones, each with a subtending leaf having the same distinctive features as the sterile foliage such as twisted bases and thickened transfusion-tissue bands (Wilf et al. 2017b: figs 61–68), is entirely dissimilar to the reproductive organs of any grasses.

The evidence here gathered firmly supports combining *Chusquea oxyphylla* and *Retrophyllum spiralifolium* into *Retrophyllum oxyphyllum* comb. nov., thus preserving the priority of the older name. Additionally, the species description for the new combination is amended to accommodate additional foliage forms and a peduncle of pollen cones that, along with the distichous foliage form, are all considered to represent a single source species and placed in *R. spiralifolium* as justified by Wilf et al. (2017b) based on detailed comparisons of a sample of 82 specimens. These additional fossils and their characters provide a far more complete whole-plant understanding of the ancient species than does the lone holotype. The nomenclatural change does not affect the other two fossil *Retrophyllum* species from South America, *R. superstes* Wilf, 2017 from the terminal Cretaceous of the Lefipán Formation in Chubut, Argentina, and *R. araucoensis* (E.W. Berry) Wilf, 2017 from the Eocene Concepción–Arauco Coal Measures of Chile (Berry 1922; Florin 1940; Greenwood 1987; Wilf et al. 2017b).

## Concluding remarks

This revision of a putative *Chusquea* fossil to the podocarp genus *Retrophyllum* removes the last fossil evidence still cited for bamboos in Gondwana (see Introduction). The remaining South American bamboo fossils (see Introduction) are post-Gondwanan and contemporary with the emergence of the Isthmus of Panama. However, phylogeographic data still support a Gondwanan origin of grasses and, in some reports, bamboos in particular (see Clark et al. 1995; Clark 1997b; Bremer 2002; Bouchenak-Khelladi et al. 2010; Hodkinson et al. 2010; Soreng et al. 2017).

The deletion of a living New World genus (*Chusquea*) from the overall floral list for Eocene Patagonia further weakens the New World biogeographic signal of the late-Gondwanan vegetation of South America (see Introduction), which is currently understood to have much stronger links to the tropical West Pacific as discussed extensively elsewhere (e.g., Wilf et al. 2009, 2014, 2019; Gandolfo et al. 2011). Interestingly, when considering the full suite of specimens (Wilf et al. 2017b), *Retrophyllum oxyphyllum* preserves morphological evidence for affinity to both Old and New World living species of *Retrophyllum*. Several of its features are only found among the Old World species, such as wide bands of transfusion tissue and the presence of scale leaves and non-distichous foliage forms, whereas its lateral resin canals and acuminate leaf apices are only seen today in South American *Retrophyllum* species (see Wilf et al. 2017b).

The strongest New World signal remaining in Eocene Patagonia based on well-described macrofossils comes from fossil fruits of *Physalis* (Solanaceae), an entirely American genus (Wilf et al. 2017a). Other fossilized genera from Eocene Patagonia with New World living relatives are, like *Retrophyllum*, disjunct with the Old World today, including *Dicksonia* (Dicksoniaceae: Central and South America, Australasia, Malesia; Berry 1938; Carvalho et al. 2013), *Podocarpus* (Podocarpaceae: Africa, South and Central America, Australasia, East Asia; Berry 1938), *Orites* (Proteaceae: South America and Australia; Romero et al. 1988; González et al. 2007), and basal Asteraceae with closest living relatives in South America and Africa (Barreda et al. 2010, 2012).

## Supplementary Material

XML Treatment for Retrophyllum oxyphyllum
